# Evaluation of the Effects of Synovial Multipotent Cells on Deep Digital Flexor Tendon Repair in a Large Animal Model of Intra‐Synovial Tendinopathy

**DOI:** 10.1002/jor.24423

**Published:** 2019-08-16

**Authors:** Mohammad R. Khan, Roger K. Smith, Frederic David, Richard Lam, Gillian Hughes, Roberta De Godoy, Andrew J. Carr, Allen E. Goodship, Jayesh Dudhia

**Affiliations:** ^1^ Clinical Sciences and Services Royal Veterinary College Hawkshead Lane Hertfordshire AL9 7TA United Kingdom; ^2^ Writtle Agricultural College Lordship Road Chelmsford Essex CM1 3RR United Kingdom; ^3^ Botnar Research Centre, Institute of Musculoskeletal Sciences University of Oxford Oxford OX3 7LD United Kingdom; ^4^ UCL Institute of Orthopaedics and Musculoskeletal Science (IOMS) Stanmore HA7 4LP United Kingdom

**Keywords:** synovium, synovial multipotent cells, tendon, digital flexor tendon, ovine

## Abstract

Intra‐synovial tendon injuries are a common orthopedic problem with limited treatment options. The synovium is a specialized connective tissue forming the inner encapsulating lining of diarthrodial joints and intra‐synovial tendons. It contains multipotent mesenchymal stromal cells that render it a viable source of progenitors for tendon repair. This study evaluated the effects of autologous implantation of cells derived from normal synovium (synovial membrane cells [SMCs]) in augmenting repair in an ovine model of intra‐synovial tendon injury. For this purpose, synovial biopsies were taken from the right digital flexor tendon sheath following creation of a defect to the lateral deep digital flexor tendon. Mononuclear cells were isolated by partial enzymatic digestion and assessed for MSC characteristics. Cell tracking and tendon repair were assessed by implanting 5 × 10^6^ cells into the digital flexor tendon sheath under ultrasound guidance with the effects evaluated using magnetic resonance imaging and histopathology. Synovial biopsies yielded an average 4.0 × 10^5^ ± 2.7 × 10^5^ SMCs that exhibited a fibroblastic morphology, variable osteogenic, and adipogenic responses but were ubiquitously strongly chondrogenic. SMCs displayed high expression of CD29 with CD271^NEGATIVE^ and MHC‐II^LOW^ cell‐surface marker profiles, and variable expression of CD73, CD90, CD105, CD166, and MHC‐I. Implanted SMCs demonstrated engraftment within the synovium, though a lack of repair of the tendon lesion over 24 weeks was observed. We conclude healthy synovium is a viable source of multipotent cells, but that the heterogeneity of synovium underlies the variability between different SMC populations, which while capable of engraftment and persistence within the synovium exhibit limited capacity of influencing tendon repair. © 2019 The Authors. *Journal of Orthopaedic Research^®^* published by Wiley Periodicals, Inc. on behalf of Orthopaedic Research Society J Orthop Res 38:128–138, 2020

Mesenchymal stem cells (MSCs) have garnered much scientific interest due to their therapeutic potential in regenerative medicine and tissue engineering. While lacking a definitive identity, MSCs are widely acknowledged to exhibit (i) tissue culture plastic adherence; (ii) spindle‐shaped fibroblastic morphology; (iii) the ability to form single cell‐derived colonies; (iv) high proliferative rate; (v) potential for osteogenic, adipogenic, and chondrogenic lineage specification; and (vi) the expression of specific cell surface epitopes.^1^, ^2^ These defining criteria have been further extended to include specific gene expression profiles conferring MSCs with phenotypic multipotency,^3^ as well as the production of paracrinal immunomodulatory trophic factors.^4^, ^5^ While MSCs have been isolated from different tissues including synovium, bone marrow, and adipose tissue remain the predominantly common sources due to ease of tissue sampling and isolation of cells from these tissues.^2^

The synovium is a specialized connective tissue that covers exposed soft tissues within diarthrodial joints, tendon sheaths, and bursae.^6^ It is generally composed of two layers; a continuous layer overlying tissues composed of one or two sheets of fibroblastic and macrophage‐like synoviocytes called the intima, and an underlying collagenous extracellular matrix containing fibroblastic synoviocytes with blood and lymphatic vessels called the sub‐intima.^2^, ^6^ Additionally, the synovium has been shown to contain multipotent MSCs with characteristics similar to bone marrow MSCs,^7^, ^8^ but reportedly with a superior chondrogenic potential,^9^ and capable of engrafting from the synovium into tendon matrices in an ex vivo model.^10^

Intra‐synovial tendinopathies, such as rotator cuff tears of the shoulder, which are frequently associated with rupture through the overlying synovial capsule and therefore exposed to synovial fluid, pose a significant socio‐economic burden complicated further by limited effective treatment options. Frequently injury results in “failed healing” where the tendon integrity has not been restored. On the basis of encouraging outcomes with the use of bone marrow MSCs for the treatment of extra‐synovial naturally occurring tendinopathies in a large animal model,^11^, ^12^ we assessed the reparative efficacy of bone marrow MSC implantations on repairing intra‐synovial tendon lesions using an ovine large animal model that mimics naturally occurring intra‐synovial tendon disease in humans and horses more accurately than extra‐synovial tendon locations in small animal models; including similar (compressive) biomechanical environment, an intra‐synovial location, and failure to heal with persistent pain. We hypothesized that implanted cells could either seal the defect from the synovial environment and/or participate in regenerative tissue repair. However, autologous bone marrow MSC implantation does not augment intra‐synovial tendon repair where these cells only engraft and persist within synovial tissue.^13^

It is not clear if bone marrow‐derived MSCs could replace the functional or reparative capacities of native tissue‐specific MSCs,^2^ and so this study evaluated the efficacy of synovial MSCs in augmenting tendon repair in an ovine model of intra‐synovial tendinopathy. SMCs carry an advantage, in that they are easily retrievable from the tendon sheath synovium for use in homologous fashion (reimplantation into same location from where originally retrieved). Therefore, we hypothesized that tissue specificity of synovial MSCs with the intra‐synovial micro‐environment would enable an improved healing response compared with the ineffectiveness of bone marrow MSCs.^13^

## MATERIALS AND METHODS

### Animal Study

The study was carried out under Home Office (UK) licence (PPL 70/6964) with approval from the Ethics and Welfare Committee of the Royal Veterinary College. A total of 26 healthy adult (3–5 years of age) female English mule sheep were used. Four animals were used to assess the distribution of iron nano‐particle‐labeled cells at 1 (*n* = 2) and 2 (*n* = 2) weeks post‐implantation. An additional 22 were used to assess the effects of unlabeled cells on tendon repair after 4 (*n* = 6), 12 (*n* = 8), and 24 (*n *= 8) weeks post‐implantation. The 4‐week time point used fewer animals because it was not anticipated to observe healing this early but rather to be able to assess other effects of the implanted cells.

### Tendon Injury Model

Establishing the injury model and evaluation comprised three stages: (i) formation of a surgical lesion in the deep digital flexor tendon (DDFT), (ii) intra‐synovial implantation of cells 2 weeks post‐surgery, and (iii) euthanasia of animals for gross evaluation and histological examination at 4, 12, and 24 weeks post‐implantation. For cell tracking experiments, forelimbs were assessed by magnetic resonance imaging (MRI), gross evaluation, and histological examination at 1 and 2 weeks post‐implantation.

**Figure 1:**
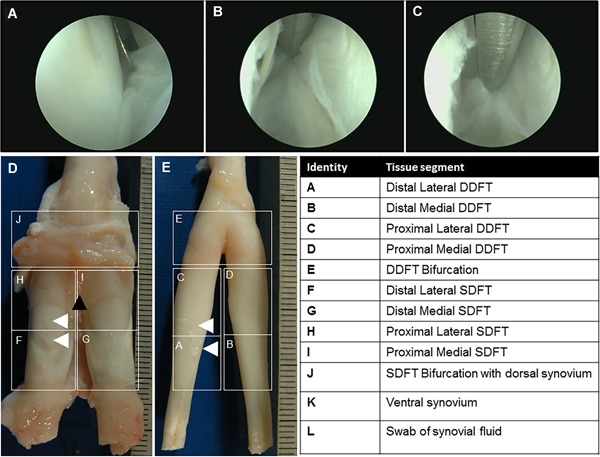
Ovine intra‐synovial digital flexor tendons. (A) Arthroscopic image of the extraction of a synovium biopsy from the digital flexor tendon sheath. (B) Dorsal view of the digital flexor tendon complex after dissection from the forelimb showing the deep digital flexor tendon (DDFT) overlaid by the superficial digital flexor tendon (SDFT). Arrow indicates the area of biopsy extraction. Arrowheads indicate lesion in the lateral deep digital flexor tendon. (C) Dorsal view of the deep digital flexor tendon with white arrowheads pointing towards the lesion. Delineations represent tissue segmentations of the DDFT and SDFT for histology that are elaborated in the accompanying table. [Color figure can be viewed at wileyonlinelibrary.com]

The surgical procedure has been detailed previously.^13^ Briefly, a linear 5 mm long by 2 mm deep defect was created in the lateral DDFT using a disposable triangle knife (ECTRA II; Smith and Nephew, UK) within the digital sheath immediately proximal to the metacarpophalangeal joint of the right forelimb by tenoscopy under general anesthesia. Simultaneously a biopsy was taken from the synovium adjacent to the tendon bifurcation proximal to the site of the lesion using basket forceps (Fig. 1A–C). Surgical portals were closed with single simple interrupted sutures of 2‐0 monofilament nylon and covered in a sterile non‐adherent dressing and bandage.

### SMC Implantation

Distal limb circumference above and below the metacarpophalangeal joint was measured to assess local inflammation. The right limb was prepared aseptically and a 23 G arterial catheter introduced into the lateral compartment of the proximal tendon sheath under ultrasound guidance, positioned immediately deep to the flexor tendons and the stylet withdrawn before 5 × 10^6^ autologous SMCs (passage 2) in 1 ml of phosphate‐buffered saline (PBS) were injected into the sheath. Accurate placement was identified by the presence of echogenic air bubbles present inside the sheath cavity post‐injection. The control group has been previously reported, which showed no healing for vehicle‐only (PBS without cells) injected into this sheep tendon injury model.^13^

Sheep received two fentanyl patches (75 μg/h; Durogesic, Aperio Technologies, Vista, CA) 12 h prior to surgery followed by a second pair 60 h post‐surgery and 0.6 mg buprenorphine (Vetergesic) 72 h later. The sheep were individually housed for 1 week and then group housed with free exercise. Lameness was assessed subjectively daily.

### Euthanasia and Post‐Mortem Analysis

Sheep were euthanized using an overdose of 20% pentobarbital (0.7 mg/kg, mean volume of 40 ml/animal). Forelimb circumference below and above the metacarpophalangeal joint was remeasured before disarticulation at the carpus for MRI or dissection for gross and histological analysis.

### Dissection and Histology

The digital flexor tendon complex was dissected out as previously described.^13^ The DDFT was separated from the superficial digital flexor tendon (SDFT) and the tissues were divided into 12 segments (Fig. 1D and E and accompanying table). Paraformaldehyde fixed segments were paraffin‐embedded to cut transverse sections using N35HR blades (Feather) at 8 μm thickness for hematoxylin and eosin (H&E) or Prussian Blue staining. Digital whole slide images of tissue sections were generated (Hamamatsu NanoZoomer s60 or a Leica SCB 400 F, Hamamatsu Photonics, Hamamatsu, Japan) and analyzed with Pathology Slide Viewing Software NDP View version 2.0 (Hamamatsu) or Aperio ImageScope version 12.1.0.5029 (Aperio Technologies).

### MRI of Sheep Forelimbs

MRI was used to track MION‐labeled cells. Isolated forelimbs were scanned from the distal aspect of the digits to the mid metacarpal region with a 1.5 T MR scanner (Philips Intera 1.5 T Pulsar System; Philips Medical Systems, Guildford, UK). Images were acquired in the transverse plane with a 61 × 61 mm field of view, 0.55 mm slice thickness, and 0.51 × 0.51 × 0.55 mm voxel size. Gradient echo sequences (Flip angle 15°, TR = 30 ms) were chosen over turbo spin echo sequences in order to maximize sensitivity to the susceptibility artifact generated by MION particles, resulting in signal void. In order to differentiate the hypointense signal generated by MION particles from other causes of signal void, such as scar tissue (or other fibrous tissue such as tendons), a dual echo technique (short TE = 7.2 ms and long TE2 = 17.4 ms) was used. This provided spatially matching image series, in which the susceptibility artifact associated with MION would increase in size in the long TE images when compared with the short TE images, while the size of other causes of signal void would not be affected.

### Cell Culture

Synovial membrane biopsies were held in 5 ml of RPMI‐1640 (Sigma‐Aldrich, Gillingham, UK) in 50 ml tubes (Falcon; Fisher Scientific, Loughborough, UK) on ice during transport to an aseptic environment. Biopsies were transferred to a 10 cm petri dish (Nunc) and minced to smaller pieces using a sterile scalpel (Swann Morton, Sheffield, UK) before mild digestion for 12 h in growth medium supplemented with 1 mg/ml collagenase type IV (Worthington, UK; cat # 4188) and 1 mg/ml of Dispase (Gibco, Fisher Scientific, Loughborough, UK) in humidified conditions (5% CO_2_, 37°C). Growth medium comprised alpha‐MEM (Gibco) supplemented with 10% fetal bovine serum (Gibco) and 1% antibiotics (Gibco). The resulting slurry was strained through a 40 µm nylon mesh (BD Biosciences, Wokingham, UK) to exclude undigested tissue and isolated cells pelleted by centrifugation at 400*g* for 7 min before re‐suspending in 5 ml of growth medium for a trypan blue haemocytometer count. Isolated synovial membrane cells (SMCs) were then seeded in a 75 cm^2^ tissue cultures flasks (Nunc) in 15 ml of growth medium and cultured in humidified conditions. Culture medium was changed after 24 h to remove debris and non‐adherent cells and thereafter after every 48 h within the initial 7 days of culture. Near confluent adherent cells were detached with 0.25% trypsin‐ethylenediaminetetraacetic acid (EDTA) (Gibco) and re‐seeded at a density of 1,000 cells/cm^2^ in 75 cm^2^ tissue culture flasks (Nunc) to expand to 5 million cells in 2 weeks. Population doubling was determined with the formula log* N*_F_−log* N*_o_)/0.301 where *N*_F_ was the final cell number and *N*_o_ was the original number of cells cultured for the specified duration.

### Labeling of Cells

Adherent SMCs were labeled with (Molday ION (MION) Rhodamine‐B, BioPal, Worcester, MA) conjugated superparamagnetic iron oxide nano‐particles according to the manufacture's guidelines (BioPal, Worcester, MA). Briefly, growth medium of cultures at 70–80% confluence was supplemented with MIONs to a final concentration of 25 µg/ml and incubated for 2 h. Labeling was compared with supplementation of protamine sulfate (Sigma‐Aldrich) to a final concentration of 100 µg/ml to enhance MION labeling. Cultures were washed twice with PBS to remove free MIONs and detached as described above.

### Colony Forming Unit Assay

Colony forming unit analysis was performed by seeding 100 cells suspended in 3 ml of growth medium on 6 cm tissue culture petri‐dishes (Nunc). Cultures were maintained in humidified conditions with bi‐weekly medium changes for 14 days and stained by 0.5% (m/v) crystal violet (Sigma‐Aldrich) in absolute methanol (Sigma‐Aldrich). Samples were scanned to a gray‐scale image on a flatbed scanner (Epson, Hemel Hempstead, UK) at 1,200 dpi and colonies counted with (ImageJ, U. S. National Institutes of Health, Bethesda, MD).

### Confocal Microscopy

Confocal microscopy was used to visualize MION uptake by seeding 5 × 10^4^ labeled cells on treated chamber slides (Nunc, Thermo Fisher Scientific, UK) for 24 h and to additionally assess persistence of label in cells after 7 days of culture. Samples were fixed in 2% paraformaldehyde (Fisher Scientific) in PBS (Gibco) and overlaid with DAPI supplemented mounting medium (Vector Laboratories, Peterborough, UK) for confocal microscopy (Leica 710 confocal microscope; Leica, Milton Keynes, UK).

### Trilineage Differentiation

Random cell samples (*n* = 7) were selected for an assessment of tri‐lineage differentiation and evaluated as previously described for bone marrow‐derived MSCs.^13^

### Flow cytometery

SMCs (*n* = 3–9, passage 2 or 3) were immunophenotypically evaluated for the expression of cell surface markers with flow cytometery according to Khan et al.^14^ Details of the antibodies used have been detailed in Khan et al. except for CD34 (clone QBEND/10, mouse antibody with reactivity to human, cat. no. SFL547PE; Bio‐Rad, Langford, UK). Briefly, near confluent MSC populations, expanded for 7 days at an initial cell seeding density of 1,000 cells/cm^2^, which were detached with a 3:2 (v/v) combination of Accutase (StemPro; Life Technologies, Paisley, UK) and EDTA (Gibco, Life Technologies, Paisley, UK) and fixed in 4% paraformaldehyde in PBS for 30 min at room temperature. The cells were washed and re‐suspended at 1 × 10^6^ cells/ml in staining buffer, comprising PBS supplemented with 10% FBS, sheep serum, goat serum (giving a final concentration of 30% serum) with 100 mg/ml bovine serum albumin (all from Sigma‐Aldrich, Dorset, UK). A 100 µl of suspension containing 1 × 10^5^ cells was plated per well of a 96‐well plate (Nunc Thermo Scientific, Paisley, UK) for each antibody and incubated for 2 h at room temperature and protected from light. The cells were then washed and re‐suspended in 1 ml of PBS in FACS tubes (12 mm x 75 mm, Thermo Fischer, Paisley, UK) for analysis on a FACS Calibur (BD Biosciences) with a threshold of a minimum of 2.5 × 10^4^ events. The cells were gated based on homogeneity in forward and side scatter properties with fluorescence intensity changes assessed against non‐labeled cells using FlowJo (version 10).

### Statistics

Quantitative data was collated in (GraphPad Prism version 7, San Diego, CA) and expressed as the mean ± SD. In vitro analyses of cellular characteristics involved different technical replicates depending on the assay outcomes and are stated in descriptions of the methods used. Trilineage differentiation was performed in three technical replicates for each SMC population. Flow cytometric analyses were performed in tandem with each SMC population in the serial analysis isolated from a different sheep. Histological whole slide imaging scans were categorically assessed for repair.

## RESULTS

### Effects of Surgical Lesion and SM Biopsy in Sheep

The tendon defect was well‐tolerated with animals not exhibiting lameness or any significant change in body mass during the experiment. There was no significant increase in limb swelling after surgical intervention or extraction of SM biopsies.

### Characteristics of SMCs

**Figure 2:**
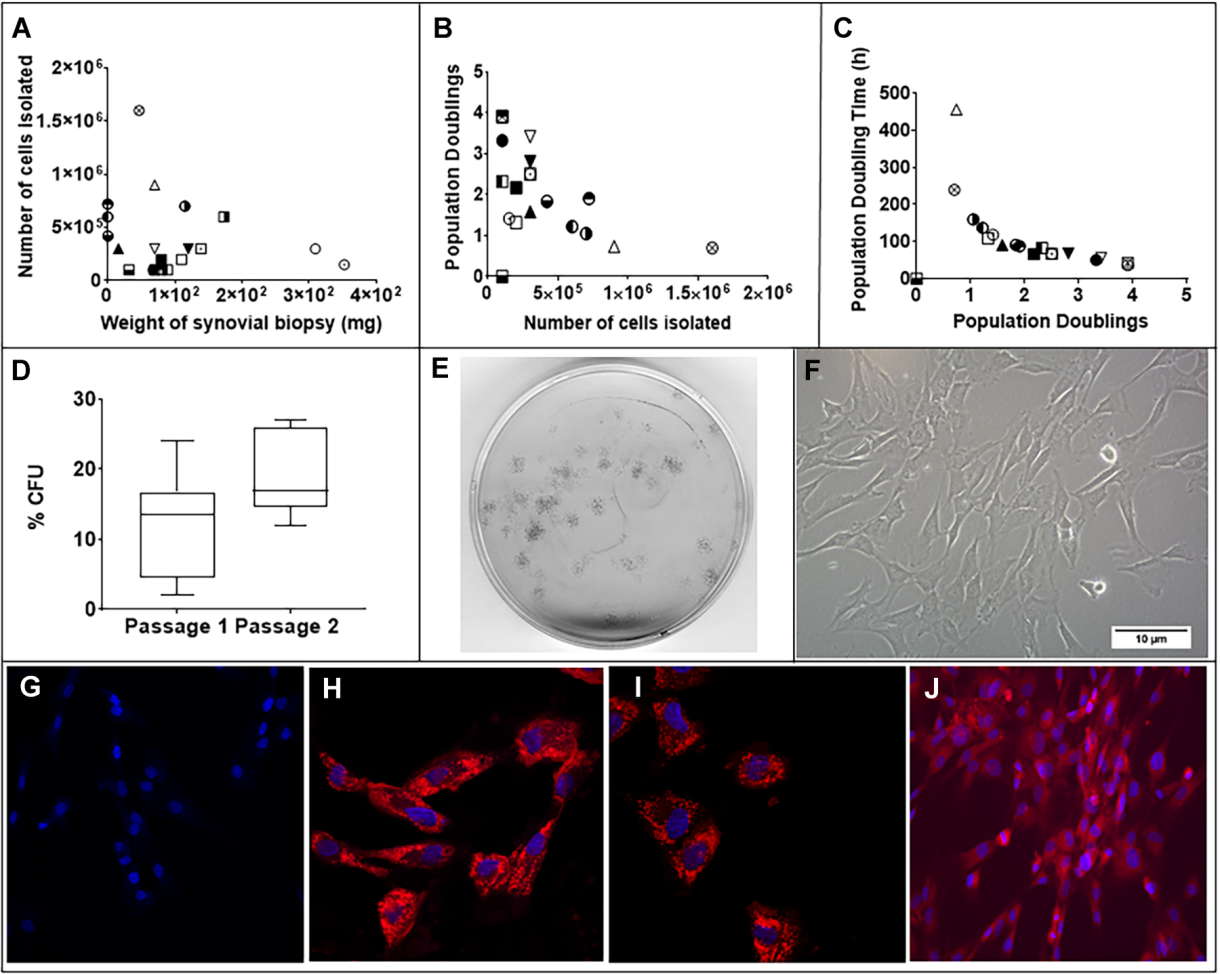
Characteristics of plastic adherent ovine synovial membrane cells. (A–C) XY scatter plot of the number of cells isolated compared with weight of synovium biopsy (A), population doublings over 7 days compared with number of isolated cells and population doubling time compared with population doublings over 7 days. Note that the biopsies occurring on the axis yielded 0.1, 0.5, and 0.2 mg of tissue. (D) Box‐whisker plot of minimum to maximum readings with median of clonogenic efficiency of cell lines (*n* = 3). (E) Scan of 6 cm petri‐dish stained for colonies with crystal violet. (F) Phase contrast microscopic image (×20 objective) of cells. (G–I) Confocal micrographs at ×40 magnification indicating cells without MION label (G), cells containing with MION label (H), cells containing MION label in presence of protamine sulfate. (J) Fluorescent micrographs at low magnification showing MION retention after 7 days of culture. [Color figure can be viewed at wileyonlinelibrary.com]

SM biopsies had an average weight of 97.0 ± 92.8 mg (*n* = 20) (Fig. 2A) yielding a mean 4.0 × 10^5^ ± 3.7 × 10^5^ cells (*n* = 20) (Fig. 2A) per biopsy. These cells adhered to tissue culture with a spindle‐like fibroblastic morphology exhibiting 2.03 ± 1.11 (*n* = 19) population doublings in the initial 7 days (passage 0–1) with a mean population doubling time of 106.8 ± 99.2 h (*n* = 19) (Fig. 2B–C). The (CFU, Colony Forming Unit) efficiency was determined to be 12.0 ± 7.0% (*n* = 12) in passage 1, which after re‐plating had increased to 19.6 ± 6.2% (*n* = 5) in passage 2 without any significant change occurring in passage 3 (data not shown). SMCs labeled for cell tracking experiments demonstrated successful uptake of MIONs, which was not enhanced by the addition of protamine sulfate (Fig. 2G–I). MION label persisted in cells cultured for 7 days (Fig. 2J) without affecting cellular proliferation (data not shown).

### Tri‐Lineage Differentiation

**Figure 3:**
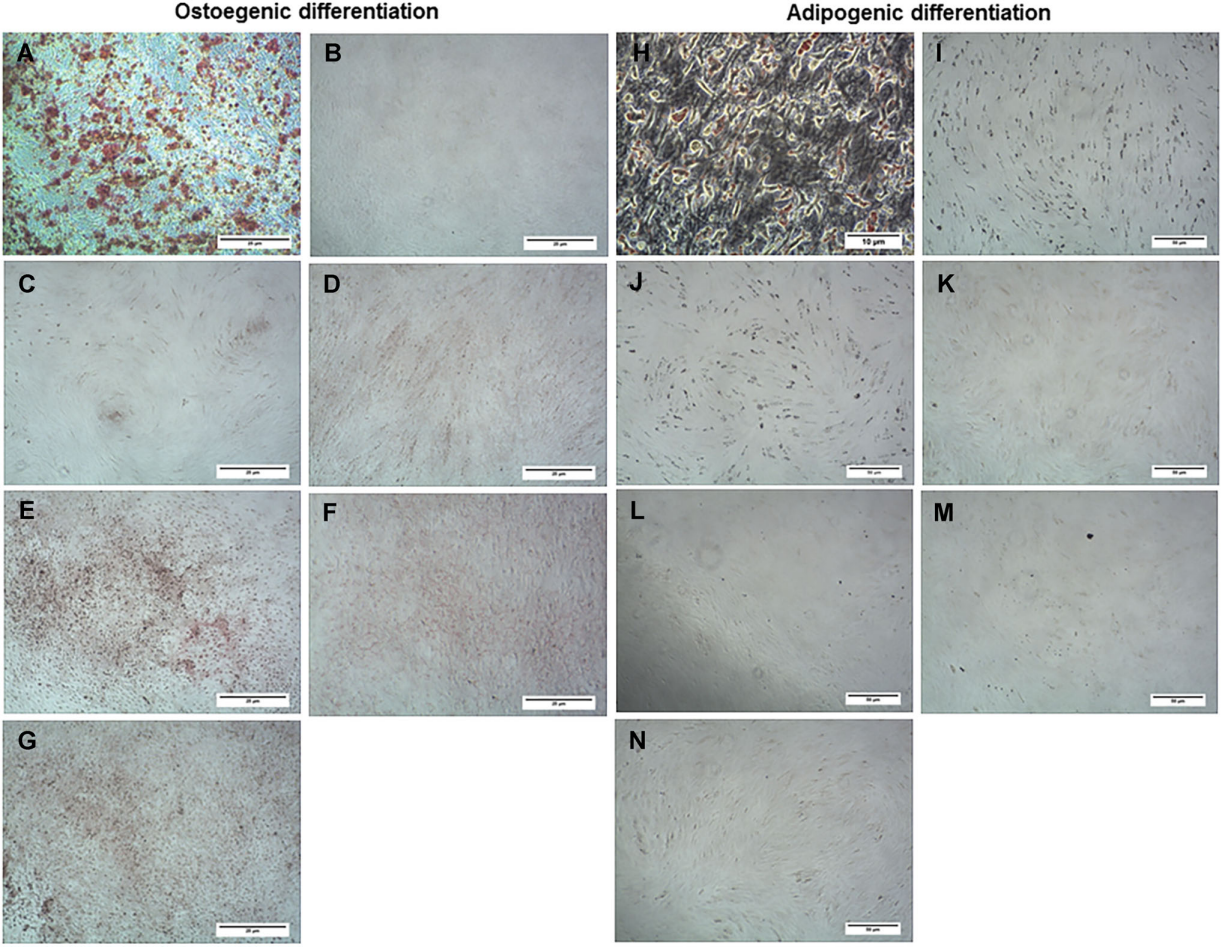
Osteogenic and adipogenic differentiation of ovine synovial membrane cells (SMCs). (A–G) Microscopic images of SMC lines (*n* = 7) after 14 days of incubation in osteogenic inductive medium, indicating variability of Alizarin Red S stain retention. (H–N) Images of Oil Red O staining of SMCs cultured in adipogenic differentiation medium for 14 days, indicating presence (H–J) and absence of staining (K–N). [Color figure can be viewed at wileyonlinelibrary.com]

**Figure 4:**
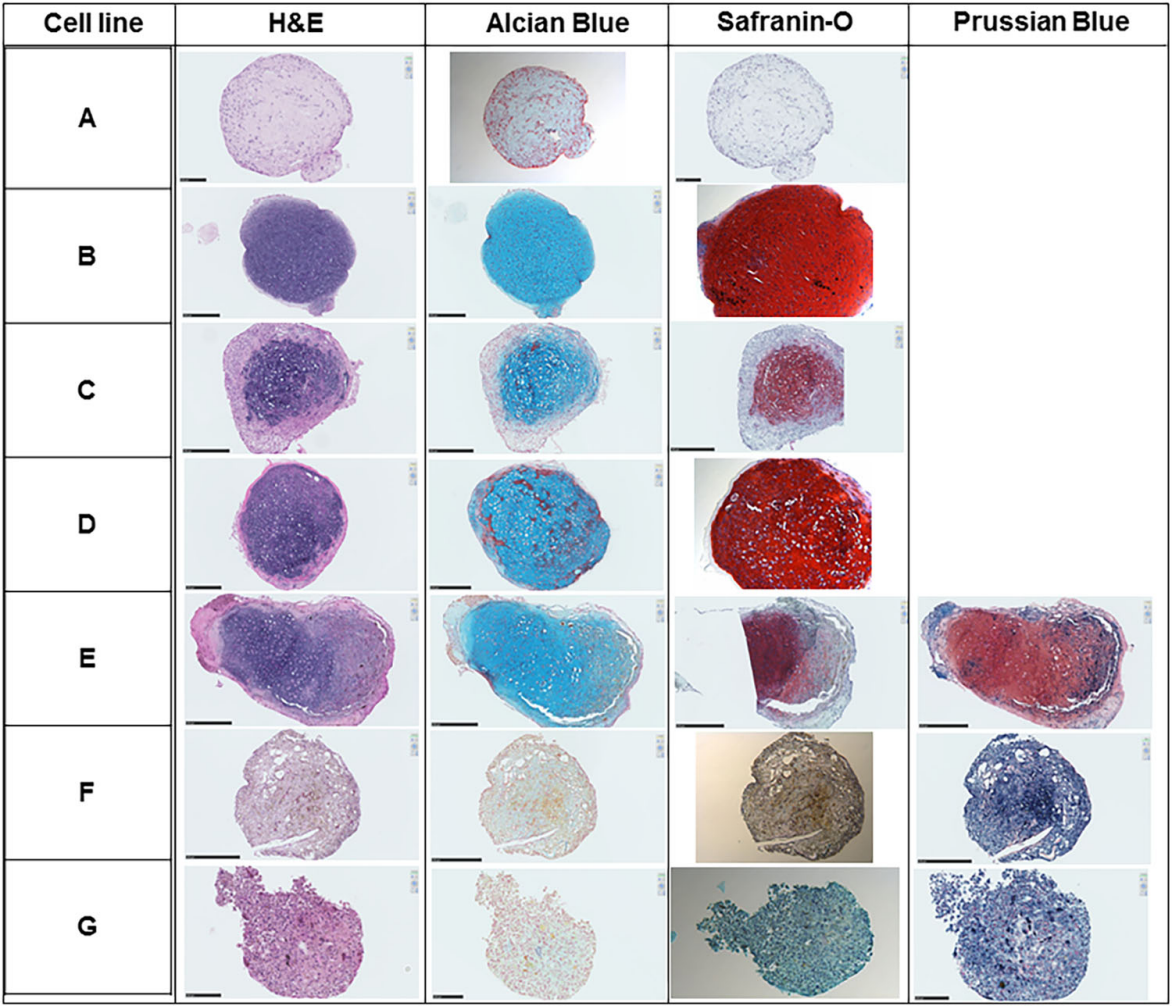
Chondrogenic differentiation of ovine synovial membrane cells (SMCs). Whole slide images of chondrogenic cell pellets after staining with hematoxylin and eosin (H&E), Alcian Blue, Safranin O, and Prussian blue. [Color figure can be viewed at wileyonlinelibrary.com]

SMCs exhibited variable osteogenic and adipogenic differentiation but were ubiquitously strongly chondrogenic. Five out of seven cell lines showed moderate Alizarin Red S staining after culture in osteogenic differentiation medium for 14 days (Fig. 3A–G). Only 3/7 of the SMCs displayed strong Oil Red O positive staining of cytoplasmic lipid vacuoles (Fig. 3H–N). All cell lines stained positive for glycosaminoglycan (GAG)‐related Alcian Blue and Safranin O staining (Fig. 4A–G). MION labeling had an inhibitory effect on osteogenic and adipogenic differentiation (data not shown) and resulted in reduced GAG staining in 2/3 cell lines tested for chondrogenic differentiation (Fig. 4E–G).

### Flow Cytometry of Cell Surface Markers

**Figure 5:**
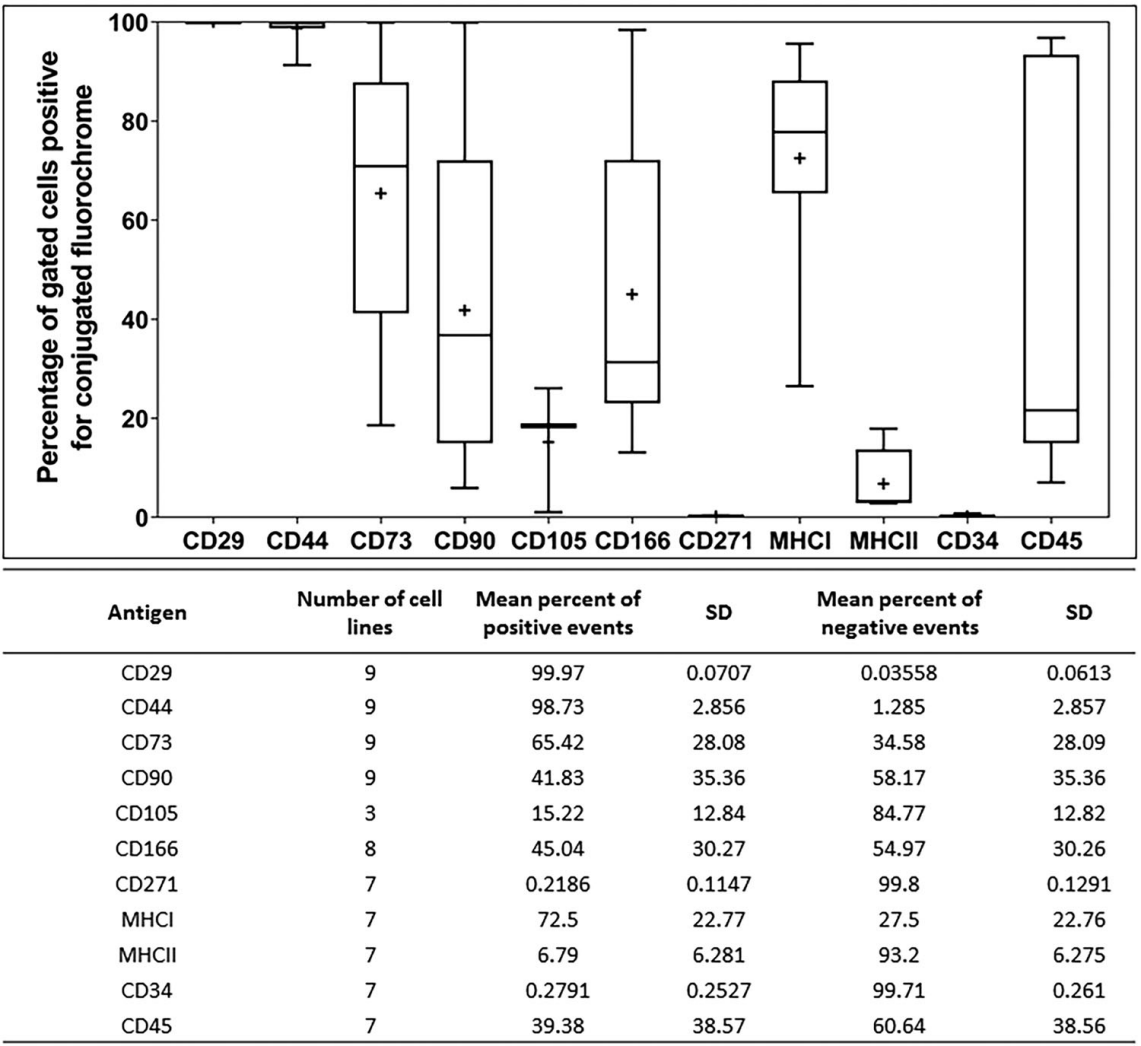
Immunophenotypic analysis of ovine synovial membrane cells (SMCs).

**Figure 6:**
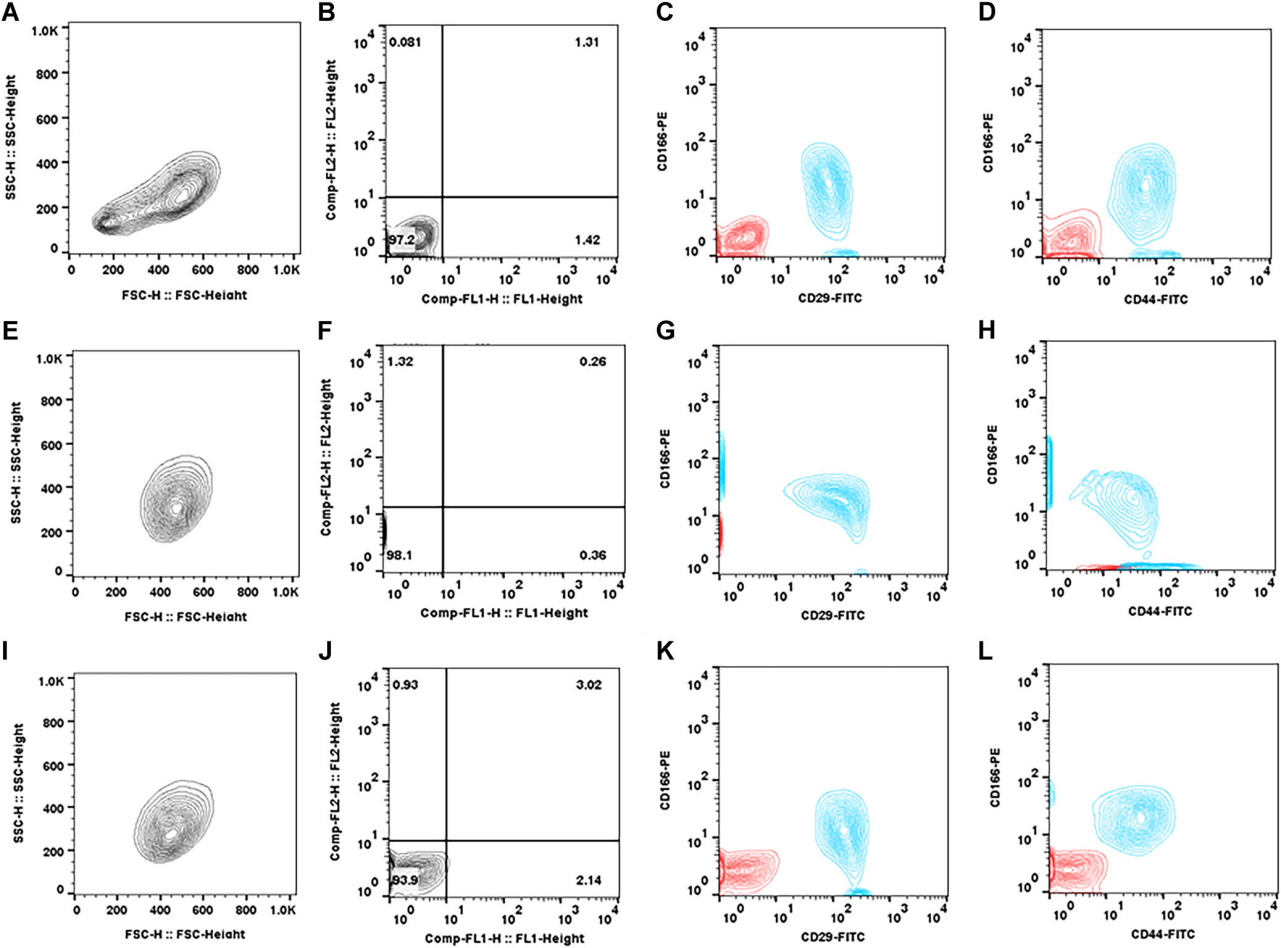
Flow cytometric analysis of dual staining assessing the co‐localization of CD166 with CD29 and CD44 in three synovial membrane cell (SMC) lines. Each of these cell lines displayed different percentages of CD166 co‐localization with CD29 and CD44 in addition to the variable expression of the marker between CD166 positive cells. [Color figure can be viewed at wileyonlinelibrary.com]

SMCs were highly variable for the expression of MSC‐related cell‐surface markers (Fig. 5). The fibroblastic markers CD29 (99.9 ± 0.0%) and CD44 (98.7 ± 2.8%) were ubiquitously detected in all cell lines tested. However, the expression of CD73 (65.4 ± 28.1%), CD90 (41.8 ± 35.4%), CD166 (45.0 ± 30.3%), and MHC‐I (72.5 ± 22.8%) were inconsistent between the different cell lines, which also exhibited low numbers of CD105 (15.3 ± 12.8%) positive cells. A dual stain experiment (*n* = 3) demonstrated a differential expression and co‐localization of CD166 with CD29 (68.8 ± 7.3%) and CD44 (58.5 ± 25.1%) in three SMC populations (Fig. 6). All cell lines tested negative for the expression of CD271 (0.2 ± 0.1%) and contained low levels of cells positive for monocyte markers MHC‐II (6.8 ± 6.3%) and CD34 (0.3 ± 0.3%) but had elevated levels of CD45 (39.4 ± 38.6%), possibly indicative of leukocytic contamination.

### Magnetic Resonance Imaging for Tracking MION‐Labeled Cells

**Figure 7:**
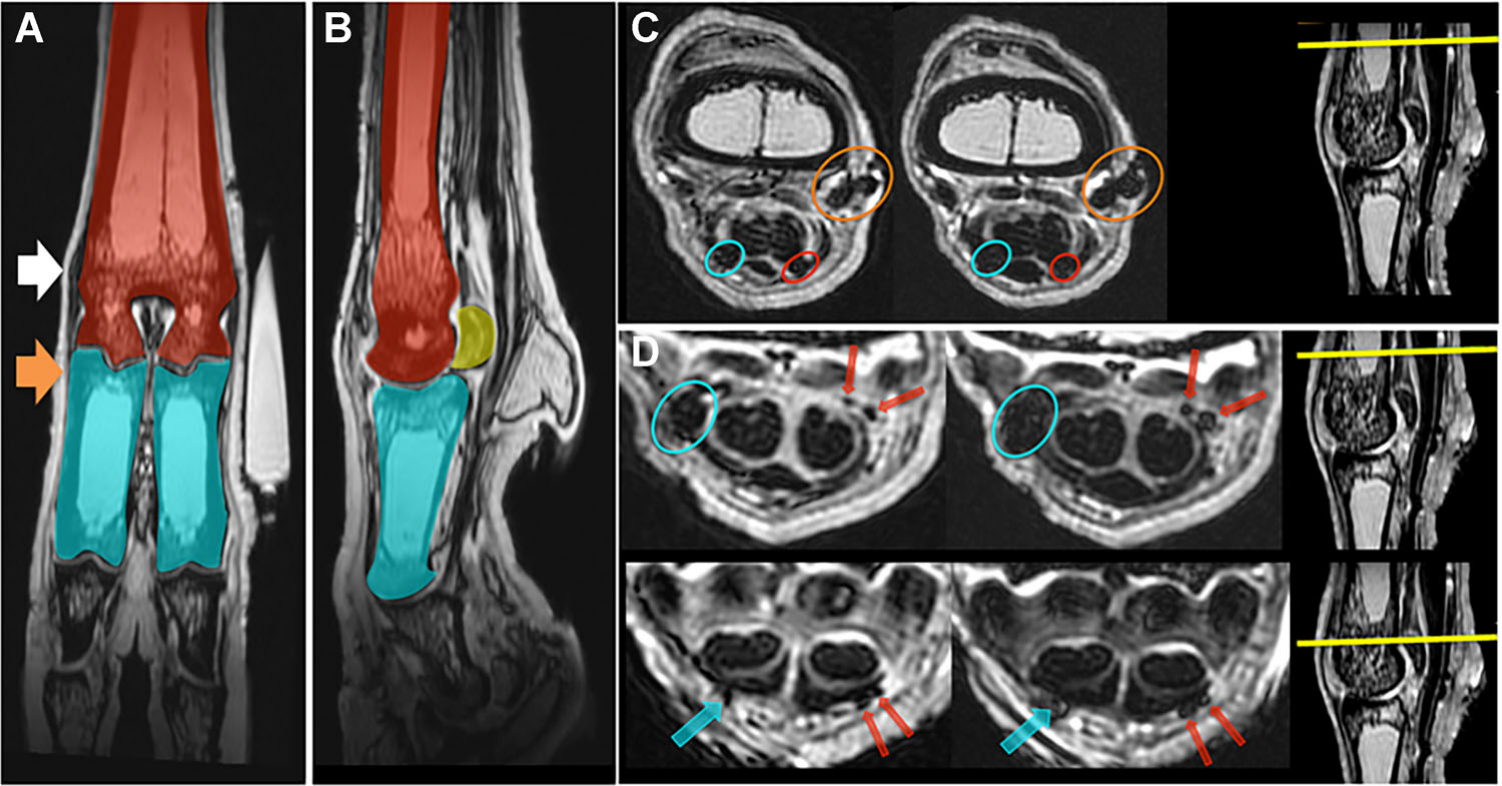
Magnetic resonance imaging (MRI) scans of distal forelimbs implanted with MION‐labeled synovial membrane cells (SMCs). Distal forelimb MRI scans along the (A) dorsal and (B) para‐sagittal planes, in which red indicates the metacarpals, blue indicates proximal phalanges, and yellow indicates proximal sesamoid bones. White arrow head indicates instrument portal and orange indicates the surgical incision, tendon lesion, and injection site. (C) Proton density (PD) and T2‐weighted (T2) transverse images at the proximal aspect of the tendon sheath, just distal to the bifurcation of the DDFTs. Multiple susceptibility artifacts comparable with MION are identified, and as expected are slightly more extensive on T2 than PD. This includes the instrument portal (orange), a moderate size area in the lateral (red), and medial (blue) aspect of the tendon sheath. (D) PD (left) and T2 (right) slightly distal to the bifurcation of the deep digital flexor tendon (DDFT) (first row) and at the apex of the proximal sesamoid bones (second row). MIONs identified along both the lateral (red) and medial (blue) aspect of the tendon sheath. [Color figure can be viewed at wileyonlinelibrary.com]

MRI indicated the presence of multiple susceptibility artifacts comparable with MION‐labeled cells in the lateral and medial aspects of the tendon sheath. The lesion was not clearly identified in these scans (Fig. 7). Some of these signals were identified in the surgical and instrument portals (Fig. 7C) while other signals seemed restricted to the digital flexor tendon sheath (Fig. 7D). MRI could not differentiate the hypointense signal of the MION‐labeled SMCs from the hypointense tendon signal and was insensitive to single or small groups of cells for quantitative assessment on cell distribution.

### Gross Parameters of the Tendon Synovial Sheath and Lesion

There was minimal inflammation of the digital flexor tendon sheath with only 2/8 sheep at 24 weeks exhibiting distension. Fluid was visible in 2/6 sheep at 4 weeks, 1/8 sheep at 12 weeks, and 2/8 sheep at 24 weeks. The lesion was macroscopically visible in all sheep at post‐mortem. The lesion in 1/6 sheep at 4 week and 5/8 sheep at 24 weeks appeared to be closed but was easily disrupted following gentle manipulation. Prolapsed fibers were visible in 2/6, 2/8, and 4/8 at 4, 12, and 24 weeks, respectively. There were no adhesions observed in any of the sheep treated with SMCs.

### Histological Assessment of the Effects of Cell Implantation

**Figure 8:**
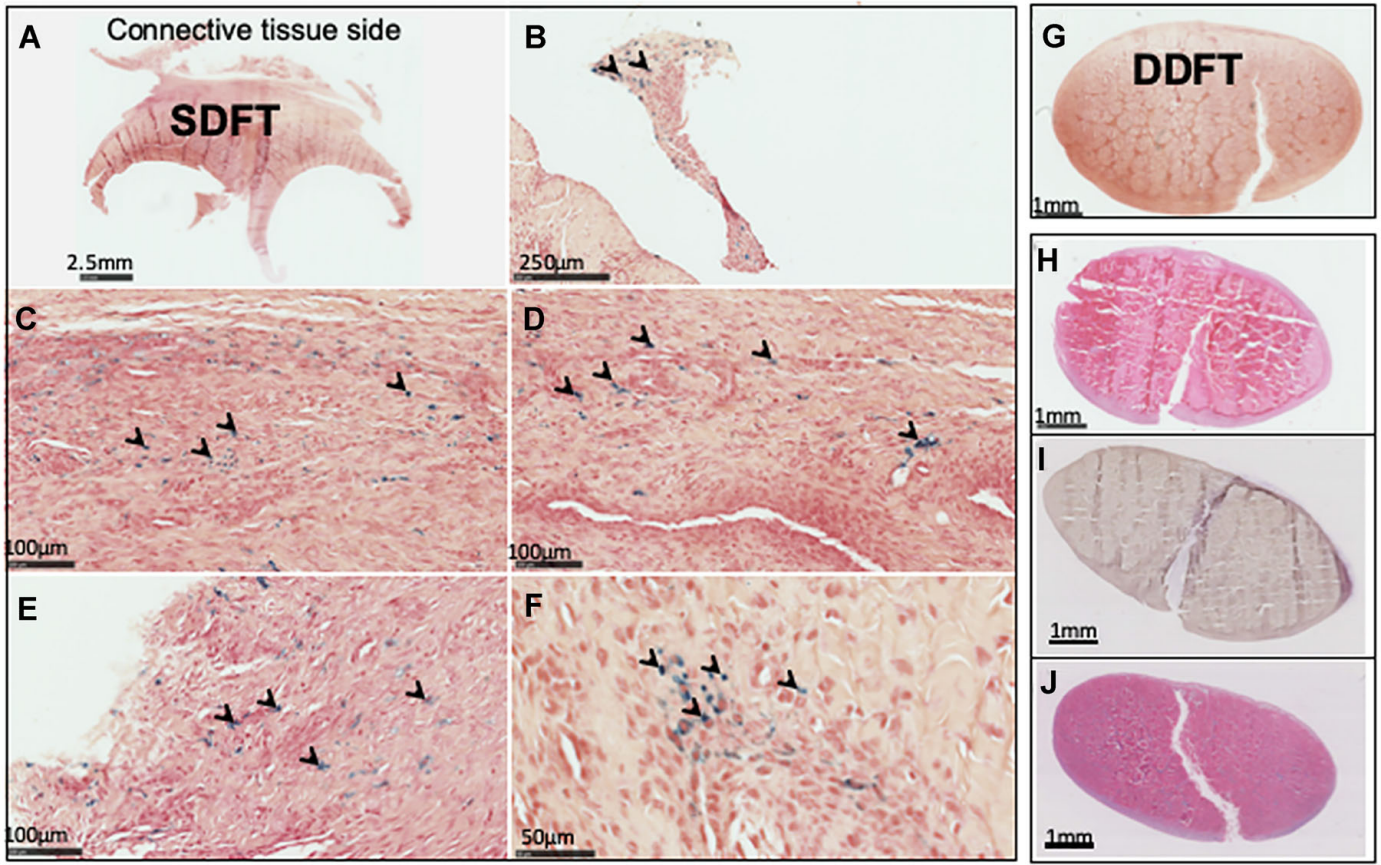
Histological assessment of ovine intra‐synovial digital flexor tendons. (A) Transverse section of synovial tissue overlying superficial digital flexor tendon (SDFT) at the tendon bifurcation site (segment J). (B–F) High magnification images of Prussian Blue stained cells (arrowheads) within the synovial tissue. (G) Transverse section of deep digital flexor tendon (DDFT) showing lesion at 2 weeks post‐implantation but absence of Prussian Blue staining. (H–J) Transverse sections of DDFTs after 4 (H), 12 (I), and 24 (J) weeks post‐implantation, indicating persistence of surgical lesion. [Color figure can be viewed at wileyonlinelibrary.com]

Histological examination of sheep treated with MION‐labeled SMCs indicated prussian blue positive cells within the palmar synovial tissue (segment J in Fig. 1E) on the palmar surface of the SDFT (Fig. 8A–F) and the dorsal synovial tissue (segment K in Fig. 1D), which was the opposing synovial tissue to the DDFT. However, labeled cells were neither within the synovial covering of the DDFT nor the tendon lesion (Fig. 8G). Prussian blue positive cells were detected as non‐homogeneous clusters engrafted within the synovium and distinct from iron associated with cells within the blood vessels (Fig. 8B–F). Histological evaluation of transverse sections of the deep digital flexor tendon in sheep treated with non‐labeled MSCs showed the persistence of the tendon lesion in all groups at 4, 12, and 24 weeks (Fig. 8H–J). These included those tendons that appeared closed on gross examination, indicating an absence of tendon healing.

## DISCUSSION

In spite of the closer relationship to injured intra‐synovial tendon tissue (synovial cells line intact tendons within a tendon sheath), this study demonstrated that SMCs, like bone marrow‐derived MSCs, are incapable of engrafting into the tendon defect and do not enhance healing of an intra‐synovial tendon injury in this sheep model of tendinopathy, although they are able to engraft into synovium. No adhesions were found after implantation of SMCs, which is a common consequence of intra‐synovial tendon healing in some locations (e.g., the hand). One limitation of this study was the absence of quantitative assessment of tendon function, such as mechanical properties. However, this model creates a longitudinal split in the tendon, which is unlikely to significantly influence the tendon's mechanical properties. Furthermore, the goal was to assess the potential of SMCs to either seal the defect from the synovial environment and/or participate in regenerative repair. A major concern with tears communicating with the synovial environment is persistent inflammation due to the shedding of tendon extracellular matrix components into the synovial cavity. Hence, the ability of SMCs to engraft and seal the defect would have a major therapeutic benefit.

The SMCs recovered from normal tendon sheath synovium were found to be a heterogeneous population of cells exhibiting differences in proliferation, clonogenicity, multipotentiality, and cell‐surface marker expression. While displaying an overall spindle‐like fibroblastic morphology similar to bone marrow‐derived MSCs, SMCs yielded substantially lower number of CFUs compared with percentages commonly reported for bone marrow MSCs.^13^, ^15^ The highly variable population doubling times of SMCs also contrasted with that reported for bone marrow MSCs (106.8 ± 99.2 and 32.29 ± 1.72 h, respectively).^13^ Osteogenic and adipogenic responses also varied but appeared not be related to tissue biopsy cell yield or cell proliferation data. Chondrogenic differentiation was ubiquitously strong as shown previously for synovium‐derived cells,^9^ and was irrespective of biopsy size, initial number of isolated cells, or capacity of osteogenic/adipogenic differentiation. These observations infer that the isolated SMC populations had a propensity toward a chondrogenic phenotype, which has also been reported for ovine MSCs recovered from synovial fluid.^16^

Flow cytometry revealed a highly variable cell‐surface marker profile between SMCs isolated from different biopsies. While the abundance of multipotent cells within healthy synovium is reportedly lower than in pathological synovium such as osteoarthritis, it may also differ between sub‐anatomical locations as demonstrated for inflammed synovium from osteoarthritic knee joints.^17^, ^18^ It is likely that the digital synovium has similar complex sub‐tissues that contribute to the observed heterogeneity. The impact on SMC phenotypic characteristics from their synovial, perivascular, or fibrous connective origin, as well as the inflammatory state of the synovium from which they were derived, may significantly influence the outcomes of regenerative approaches for tendon repair.^19^ A relevant example is the absence of expression of cell‐surface marker CD271, a low affinity neural growth factor receptor reported to be a marker of multipotent cells; while largely absent in normal healthy synovium cells it is upregulated in pathologic inflammed synovial tissue.^2^, ^20^, ^21^ Similarly, SMC characteristics appear profoundly influenced by joint type with knee‐derived tissue deemed a preferable source for multipotent cells than the hip joint due to the stronger osteogenic and adipogenic differentiation responses of cells,^22^ which may further vary for synovium located in tendon sheaths.

While cell‐surface marker expression showed consistent high expression for two robust MSC markers, CD29 and CD44, there was a wide range in expression for CD73, CD90, CD166, and MHC‐I. CD105 was expressed at lower levels compared with our observations with ovine bone marrow MSCs,^13^, ^14^ and an absence of CD271 expression, which is consistent with reports in normal human joint synovium.^21^, ^23^, ^24^ While the hematopoietic marker CD34 was absent, there was variable expression of CD45 and MHC‐II, indicating potential inclusion of leukocytic cells from perivascular tissue. However, these profiles are not dissimilar to recent studies of synovial MSCs derived from inflammed human knee joints.^18^, ^21^, ^22^, ^23^, ^25^, ^26^, ^27^ Hagmann et al.^25^ reported differences between donor‐typed bone marrow and synovial MSCs with the latter uniformly exhibiting trilineage differentiation and strongly positive for CD73, CD90, CD105, and MHC‐I although small levels of CD34 and CD45 positive cells were also detected. A comparable yield of cells from synovium have been isolated from pathological knee and hip joints exhibiting a “mixed” cell‐surface marker profile similar to this study.^22^ Furthermore, a similar cell‐surface marker expression profile for synovium‐derived trilineage positive cells has been reported in this study, though the authors have interpreted CD166 as a possible discriminating factor between multipotent and fibroblastic cells.^26^ In contrast, assessing the ameliorative effects of synovial MSCs in an osteoarthritic mouse model, synovial MSCs were reported to be highly positive for CD73, CD90, and CD105.^27^ The lack of CD105 and CD271 with moderate CD73 positive cells was reported for the surface and stromal synovia from human arthritic joints.^18^ These findings correspond to the SMC heterogeneity seen in this study supporting our view that the variability is likely due to the inclusion of stromal and perivascular synovial tissue in biopsies. It may be possible to reduce this heterogeneity by applying retrieval methods such as those recently described for retrieving minimally manipulated SMCs from joints with greater cellular homogeneity.^28^ It remains to be established whether such cell populations are more amenable to effect tendon repair although our observations suggest that none of the subsets are capable of engrafting in the tendon defect.

The results of this study indicated an inverse correlation between the number of SMCs isolated and tissue weight, and a trend that corresponded with population doubling and population doubling time suggesting that smaller biopsies likely yielded a greater abundance of proliferative spindle‐shaped SMCs. Although we did not extend the population doubling experiments until exhaustion of proliferative capacity, a small decrease in CFU ability upon re‐plating SMCs for the second passage was observed.

The ovine SMCs in this study displayed engraftment and migration within the synovium as a result of homologous implantation, appearing as homogeneously distributed clusters within synovial tissue though failing to adhere the intact tendon surface or the lesion, similar to bone marrow‐derived MSCs.^13^ This selective engraftment of cells to the synovium, although in small numbers as previously shown for MSCs,^13^ and lack thereof to the tendon suggests that synovium is the preferred niche for these cells. It therefore remains to be established whether SMCs or other multipotent cells can be applied successfully for tendon repair for injuries that occur within the synovium sheath. We propose the absence of healing within this compartment, in addition to the innate inability for synovially located tendons to repair, is due to the infiltration of the synovial fluid into the injured tendon matrix, which we have previously shown to be toxic to both MSCs and tendon fibroblasts,^29^ and that intra‐synovially injected cells are not able to seal the tendon lesion from the synovial environment because of their lack of adherence to tendon matrix as well as poor adherence to the tendon surface. Future work elucidating the underlying mechanism of cellular engraftment and migration within the synovium could allow the directed homing of MSCs to tendon repair sites or enhancement of cell retention such as with scaffolds as described for electrospun nanofiber sutures in a rat model of rotator cuff ruptures,^30^ thereby significantly contributing to the effectiveness of therapeutic strategies for intra‐synovial tendon repair.

## CONCLUSION

The healthy synovium in tendon sheaths is a viable source of multipotent progenitor cells. The abundance of these cells is variable; however, despite their heterogenous nature, SMCs show similar engraftment and persistence within synovium but not within intact tendon or tendon lesions. Although the SMC populations did not augment tendon healing as assessed in this study, further understanding of the mechanisms of engraftment could lead to more successful cellular therapies for intra‐synovial tendon repair.

## AUTHORS’ CONTRIBUTION

The study was conceived by R.K.W.S., A.J.C., J.D., and A.E.G. R.K.W.S., R.D.G., and G.H. performed the surgeries. F.D. and R.L. performed MRI scans. M.R.K. conducted the cell culture work, flow cytometery, and whole slide imaging. M.R.K. and J.D. performed dissections, gross histological assessments of tissues, and histological analyses. M.R.K. wrote the draft of the manuscript and revised it. R.K.W.S. and J.D. revised the manuscript.

## ACKNOWLEDGMENTS

The authors would like to acknowledge the technical assistance provided by the staff of the Biological Services Unit and the Pathology services for histology of the Royal Veterinary College. The assigned manuscript approval number is CSS_02007.

## DATA AVAILABILITY

The datasets generated during and/or analyzed during the current study are available from the corresponding author on reasonable request.

## References

[1] Dominici M , Le Blanc K , Mueller I , et al. 2006 Minimal criteria for defining multipotent mesenchymal stromal cells. The International Society for Cellular Therapy position statement. Cytotherapy 8:315–317. 16923606 10.1080/14653240600855905

[2] de Sousa EB , Casado PL , Neto VM , et al. 2014 Synovial fluid and synovial membrane mesenchymal stem cells: latest discoveries and therapeutic perspectives. Stem Cell Res Ther 5:1–6. 25688673 10.1186/scrt501 PMC4339206

[3] Boeuf S , Richter W . 2010 Chondrogenesis of mesenchymal stem cells: role of tissue source and inducing factors. Stem Cell Res Ther 1:31. 20959030 10.1186/scrt31 PMC2983444

[4] Caplan AI , Dennis JE . 2006 Mesenchymal stem cells as trophic mediators. J Cell Biochem 98:1076–1084. 16619257 10.1002/jcb.20886

[5] Meirelles Lda S , Fontes A , Covas D , et al. 2009 Mechanisms involved in the therapeutic properties of mesenchymal stem cells. Cytokine Growth Factor Rev 20:419–427. 19926330 10.1016/j.cytogfr.2009.10.002

[6] D. smith M . 2011 The normal synovium. Open Rheumatol J 5:100–106. 22279508 10.2174/1874312901105010100 PMC3263506

[7] De Bari C , Dell’accio F , Tylzanowski P , et al. 2001 Multipotent mesenchymal stem cells from adult human synovial membrane. Arthritis Rheum 44:1928–1942. 11508446 10.1002/1529-0131(200108)44:8<1928::AID-ART331>3.0.CO;2-P

[8] Jones EA , Crawford A , English A , et al. 2008 Synovial fluid mesenchymal stem cells in health and early osteoarthritis: detection and functional evaluation at the single‐cell level. Arthritis Rheum 58:1731–1740. 18512779 10.1002/art.23485

[9] Sakaguchi Y , Sekiya I , Yagishita K , et al. 2005 Comparison of human stem cells derived from various mesenchymal tissues: superiority of synovium as a cell source. Arthritis Rheum 52:2521–2529. 16052568 10.1002/art.21212

[10] Hayashi M , Zhao C , An KN , et al. 2012 Cell migration after synovium graft interposition at tendon repair site. Hand 7:374–379. 24294156 10.1007/s11552-012-9453-x PMC3508017

[11] Smith RKW , Werling NJ , Dakin SG , et al. 2013 Beneficial effects of autologous bone marrow‐derived mesenchymal stem cells in naturally occurring tendinopathy. PLoS ONE 8:1–14. 10.1371/journal.pone.0075697 PMC3783421 24086616

[12] Godwin EE , Young NJ , Dudhia J , et al. 2012 Implantation of bone marrow‐derived mesenchymal stem cells demonstrates improved outcome in horses with overstrain injury of the superficial digital flexor tendon. Equine Vet J 44:25–32. 21615465 10.1111/j.2042-3306.2011.00363.x

[13] Khan MR , Dudhia J , David FH , et al. 2018 Bone marrow mesenchymal stem cells do not enhance intra‐synovial tendon healing despite engraftment and homing to niches within the synovium. Stem Cell Res Ther 9:169. 29921317 10.1186/s13287-018-0900-7 PMC6009051

[14] Khan MR , Chandrashekran A , Smith RKW , et al. 2016 Immunophenotypic characterization of ovine mesenchymal. Stem Cells Cytom Part A 89:443–450. 10.1002/cyto.a.22849 27077783

[15] Sekiya I , Larson BL , Smith JR , et al. 2002 Expansion of human adult stem cells from bone marrow stroma: conditions that maximize the yields of early progenitors and evaluate their quality. Stem Cells 20:530–541. 12456961 10.1634/stemcells.20-6-530

[16] Burk J , Glauche SM , Brehm W , et al. 2017 Characterisation and intracellular labelling of mesenchymal stromal cells derived from synovial fluid of horses and sheep. Vet J 222:1–8. 28410670 10.1016/j.tvjl.2017.02.006

[17] Hermida‐Gómez T , Fuentes‐Boquete I , Gimeno‐Longas MJ , et al. 2011 Quantification of cells expressing mesenchymal stem cell markers in healthy and osteoarthritic synovial membranes. J Rheumatol 38:339–349. 21078714 10.3899/jrheum.100614

[18] Mizuno M , Katano H , Mabuchi Y , et al. 2018 Specific markers and properties of synovial mesenchymal stem cells in the surface, stromal, and perivascular regions. Stem Cell Res Ther 9:123. 29720268 10.1186/s13287-018-0870-9 PMC5930798

[19] De Bari C , Dell’accio F , Vandenabeele F , et al. 2003 Skeletal muscle repair by adult human mesenchymal stem cells from synovial membrane. J Cell Biol 160:909–918. 12629053 10.1083/jcb.200212064 PMC2173757

[20] De Bari C , Dell’accio F , Karystinou A , et al. 2008 A biomarker‐based mathematical model to predict bone‐forming potency of human synovial and periosteal mesenchymal stem cells. Arthritis Rheum 58:240–250. 18163504 10.1002/art.23143

[21] Karystinou A , Dell’accio F , Kurth TBA , et al. 2009 Distinct mesenchymal progenitor cell subsets in the adult human synovium. Rheumatology 48:1057–1064. 19605375 10.1093/rheumatology/kep192

[22] Hatakeyama A , Uchida S , Utsunomiya H , et al. 2017 Isolation and characterization of synovial mesenchymal stem cell derived from hip joints: a comparative analysis with a matched control knee group. Stem Cells Int 2017:1–13. 10.1155/2017/9312329 PMC5237455 28115945

[23] Van Landuyt KB , Jones EA , McGonagle D , et al. 2010 Flow cytometric characterization of freshly isolated and culture expanded human synovial cell populations in patients with chronic arthritis. Arthritis Res Ther 12:R15. 20105279 10.1186/ar2916 PMC2875643

[24] Toyoda E , Sato M , Takahashi T , et al. 2019 Multilineage‐differentiating stress‐enduring (Muse)‐like cells exist in synovial tissue. Regen Ther 10:17–26. 30525067 10.1016/j.reth.2018.10.005 PMC6260259

[25] Hagmann S , Rimmele C , Bucur F , et al. 2016 Mesenchymal stromal cells from osteoarthritic synovium are a distinct population compared to their bone‐marrow counterparts regarding surface marker distribution and immunomodulation of allogeneic CD4+ T‐cell cultures. Stem Cells Int 2016:1–17. 10.1155/2016/6579463 PMC4969547 27516777

[26] Fernandes TL , Kimura HA , Pinheiro CCG , et al. 2018 Human synovial mesenchymal stem cells good manufacturing practices for articular cartilage regeneration. Tissue Eng Part C Methods 24:709–716. 10.1089/ten.tec.2018.0219 PMC6306653 30412046

[27] Yin L , Wu Y , Yang Z , et al. 2018 Microfluidic label‐free selection of mesenchymal stem cell subpopulation during culture expansion extends the chondrogenic potential in vitro. Lab Chip 18:878–889. 29459915 10.1039/c7lc01005b

[28] Baboolal TG , Khalil‐Khan A , Theodorides AA , et al. 2018 A novel arthroscopic technique for intraoperative mobilization of synovial mesenchymal stem cells. Am J Sports Med 46:3532–3540. 30419170 10.1177/0363546518803757 PMC6282154

[29] Garvican ER , Salavati M , Smith RKW , et al. 2017 Exposure of a tendon extracellular matrix to synovial fluid triggers endogenous and engrafted cell death: a mechanism for failed healing of intrathecal tendon injuries. Connect Tissue Res 58:438–446. 27726447 10.1080/03008207.2016.1245726

[30] Peach MS , Ramos DM , James R , et al. 2017 Engineered stem cell niche matrices for rotator cuff tendon regenerative engineering. PLoS ONE 12:e0174789. 28369135 10.1371/journal.pone.0174789 PMC5378368

